# Factors affecting survival in patients with endobronchial malignant mass after flexible Bronchoscopic cryotherapy: a cohort study

**DOI:** 10.1186/s12890-019-0854-2

**Published:** 2019-05-24

**Authors:** Fu-Tsai Chung, Chun-Liang Chou, Yu-Lun Lo, Chih-Hsi Kuo, Tsai-Yu Wang, Chun-Hwa Wang, Hung-Yu Huang, Horng-Chyuan Lin, Chih-Hao Chang, Chung-Shu Lee, Hao-Cheng Chen, Shu-Min Lin

**Affiliations:** 1 Department of Thoracic Medicine, Saint Paul’s Hospital, Taoyuan, Taiwan; 2 Department of Thoracic Medicine, Chang Gung Memorial Hospital at Linkou, Chang Gung University, College of Medicine, 199 Tun Hwa N. Rd, Taipei, Taiwan; 3 Graduate Institute of Clinical Medical Sciences, College of Medicine, Chang Gung University, Taoyuan City, Taiwan; 4 Department of Thoracic Medicine, Taipei Medical University Hospital, Taipei Medical University, Taipei, Taiwan; 5 Department of Thoracic Medicine, Shuang Ho Hospital, Taipei Medical University, New Taipei, Taiwan

**Keywords:** Cohort, Cryotherapy, Malignant endobronchial mass, Chemotherapy, Survival

## Abstract

**Background:**

Malignant endobronchial mass (MEM) has poor prognosis, cryotherapy is reportedly to diagnose MEM, however, the therapeutic role of cryotherapy impacts on survival has not be well addressed.

**Methods:**

Cohort data on post-cryotherapy MEM patients in a university-affiliated hospital between 2007 and 2012 were evaluated. Factors that impact survival of these subjects were analyzed using multivariate regression analysis.

**Results:**

During study period, 67 patients (47 males), with median age was 63 years (range, 50–77 and median performance status of 2 (inter-quartile range [IQR], 2–3). Twenty-five had primary lung squamous cell carcinoma, 14 primary had lung adenocarcinoma, seven had metastatic colon adenocarcinoma, four had sarcoma, four had non-small cell lung cancer, four had small cell lung cancer, three had large cell carcinoma, two had lymphoma, one had muco-epidermoid carcinoma, two had esophageal squamous cell carcinoma, and one had metastatic renal cell carcinoma. MEM were observed as follows: 15 at the trachea, 14 at the left main bronchus, 12 at the right main bronchus, 12 at the right upper lobe bronchus, five at the right intermediate bronchus, three at the right lower lobe bronchus, three at the left upper lobe bronchus, two at the left lower lobe bronchus, and one at the right middle lobe bronchus Post-cryotherapy complications included minor bleeding (*n* = 14) and need for multiple procedures (*n* = 12); outcomes were relief of symptoms (*n* = 56), improved performance status (*n* = 49) and ability to receive chemotherapy (*n* = 43). After controlling for other variables, performance status improved after cryotherapy (odds ratio [OR] 3.7; *p* = 0.03; 95% confidence interval [CI] 1.2~10.7) and ability to receive chemotherapy (OR 4.3; *p* = 0.02; 95% CI 1.4~13.7) remained significant survival factor. Patients who received chemotherapy and cryotherapy had better survival than patients who received only cryotherapy (median, 472 vs. 169 days; log-rank test, *p* = 0.02; HR 0.37; 95% CI 0.16–0.89).

**Conclusion:**

Cryotherapy could be useful management of MEM by flexible bronchoscopy. The performance status after cryotherapy improved and caused further chemotherapy possible for the study patients and thereby, improved survival. However, the mechanism in detail of cryotherapy improve survival should be explored in the future.

## Background

Patients with malignant endobronchial mass (MEM) could cause central airway obstruction (CAO) and usually have poor prognosis. Severe breathlessness and poor performance status of these patients make the rescuer treatment mandatory to resolve the condition [1–3].

CAO cause respiratory failure remains a severe complication and may result in patient mortality due to the severe narrowing of the central airway by invasion of malignant tumor [1–3]. Despite interventional bronchoscopic procedures have been reported to mitigate patient dependency, the therapy is only the palliative relief of the malignant obstruction and satisfactory results may not be immediate or lasting [4–6]. Cryotherapy by bronchoscopy to remove MEM has been reported. Cryotherapy could relieve airway obstructions by MEM both extrinsic compression and direct tumor invasion has been reported [7, 8].

Despite the use of cryotherapy to manage MEM were demonstrated, the independent effectiveness of the treatment has yet to be established in large-scale studies [9, 10]. Furthermore, few reports analyzed factors that impact on survival among post-cryotherapy MEM patients with and without further chemotherapy. Hence, this cohort study was conducted to clarify the role of cryotherapy in these patients, particularly focusing on complications and outcomes after cryotherapy and factors that impact on survival.

## Methods

### Design

This cohort study was conducted at a university-affiliated hospital in northern Taiwan. The institutional review board of the Chang Gung Medical Foundation approved the method of study, reassurance of patient privacy, and study design (IRB No.:100-3211B). All patients provided direct informed consent prior to cryotherapy.

### Patients

Between 2007 and 2012, data from sequential patients with malignant endobronchial mass who received cryotherapy by flexible bronchoscope were collected. All patients in the study were not eligible for surgical rigid bronchoscopy due to illness severity, risk or refusal of surgery.

### Baseline demography and outcomes after cryotherapy

Baseline demography with age, sex, performance status, pathology and locations of MEMs, complications, and outcomes after cryotherapy, were taken from our cohort database by records of chart. Complications included minor bleeding, major bleeding, need for multiple procedures (repeat procedures for residual mass more than two procedures), and pneumothorax. Outcomes of the pateints with MEM receive cryotherapy include relief of symptoms, improved performance status, and ability to receive further chemotherapy post-cryotherapy.

### Cryotherapy

A cryo-probe (Erbe USA, Inc., Marietta, GA) with carbon dioxide (CO_2_) as cryogen was used. A temperature of around − 70 °C was achieved at the probe tip. All study patients received cryotherapy via flexible bronchoscopy under sedation. The procedures of bronchoscopy under local anesthesia and sedation in the study followed protocols of previous reports [11–13]. We most practiced sedation under a bi-spectral index with intravenous propofol, ocassional midazolam. All patients received 2% xylocaine solution local spray and inhalation to the airway during bronchoscopy [13]. Blood pressure, electrocardiography (ECG), and oxygen saturation were monitored during bronchoscopy. During bronchoscopic procedures, the bronchoscope approached the proximal end of the lesion via a mouth guard, through the lumen of trachea and bronchi. Then, via the bronchoscope, the cryotherapy probe was inserted into airway and contacted with the main mass. Cryotherapy was applied on the main mass with -70 °C CO_2_ from 20 to 60 s. On a monitor, we confirmed that the probe and the mass were frozen. The mass was separated away by the bronchoscope after cryotherapy. After mass removal was completed, the bronchoscope was re-inserted into the airway for patency check-up.

### Assessment of factors that impact post-cryotherapy survival

Factors that had impacts on survival were evaluated. The factors included bleeding, need for multiple procedures, symptom relief, improved performance status, and ability to receive further chemotherapy after cryotherapy.

We quantified relief of symptoms (mainly breathlessness) by the Modified Medical Research Council (MRC) Scale. Level 0 is the lowest observed level of dyspnea impairment observed, and level 4 is the highest level of dyspnea impairment. The and is Medical Research Council (MRC) Scale was defined in 1999 by Bestall et al. [14] a 5-point scale based on the awareness of breathlessness felt by the patient.

The scoring system of performance status applied in this study was the Eastern Cooperative Oncology Group (ECOG) system (published by Oken et al. in 1982) [15]. The scoring system is from 0 to 5, with 0 denoting perfect health and 5 death:

ECOG score 0 – Asymptomatic (fully active, able to carry on all pre-disease activities without restriction).

ECOG score 1 – Symptomatic but completely ambulatory (restricted from physically strenuous activity but ambulatory and able to carry out work of a light or sedentary nature, for example, light housework, or office work).

ECOG score 2 – Symptomatic, < 50% in bed during the day (ambulatory and capable of all self-care but unable to carry out any work activities; up and about more than 50% of waking hours).

ECOG score 3 – Symptomatic, > 50% in bed, but not bedbound (capable of only limited self-care, confined to bed or chair 50% or more of waking hours).

ECOG score 4 – Bedbound (completely disabled; cannot carry on any self-care; totally confined to bed or chair).

ECOG score 5 – Death.

Improvement PS was defined as an ECOG score decrease from 3 or 4 to below 2; improved PS indicated the patient could receive further chemotherapy.

### Statistical analysis

Data of the study were reported as median values and inter-quartile range (IQR) or numeric values (%). To evaluate the factors impacting survival, we used the multivariate logistic regression analysis to identify the net effects of each individual factor. By logistic regression analysis, odds ratios (OR) and 95% confidence intervals (CI) were calculated to explain the influences of all supposedly independent factors after adjusting other confounding factors. Significance was defined as a *p* value less than 0.05. Survival between the two sub-groups with or without post-cryotherapy chemotherapy was compared. Survival curves were traced using the Kaplan-Meier method, and survival curves were compared using the log rank test. All analyses were conducted using SPSS software (version 13.0, SPSS, Chicago, IL) and Prism 5 for Windows (version 5.03, GraphPad Software Inc., San Diego, CA).

## Results

During the study period, 67 MEM patients received bronchoscopic cryotherapy consecutively were enrolled. Their baseline data were median age was 63 years (range, 50–77 years), the sex distribution was 47 male 20 females, and the median performance status 2 (IQR 2–3) (Table 1). Among these patients with MEM, 25 had primary lung squamous cell carcinoma, 14 primary had lung adenocarcinoma, seven had metastatic colon adenocarcinoma, four had sarcoma, four had non-small cell lung cancer, four had small cell lung cancer, three had large cell carcinoma, two had lymphoma, one had muco-epidermoid carcinoma, two had esophageal squamous cell carcinoma, and one had metastatic renal cell carcinoma.

**Table 1:** Baseline data of the study patients (*n* = 67)

Age, years	63 (50–77)
Gender, Male/Female	47/20
Performance status	2 (2–3)
Diagnosis of malignant endobronchial mass	
Primary lung SqCC	25
Primary lung adenocarcinoma	14
Colon adenocarcinoma metastasis	7
Sarcoma	4
Large cell carcinoma	3
NSCLC	4
SCLC	4
Lymphoma	2
Muco-epidermoid carcinoma	1
Esophageal SqCC invasion	2
RCC metastasis	1

*Abbreviations*: *SqCC* squamous cell carcinoma, *TB* tuberculosis, *NSCLC* non-small cell lung cancer, *SCLC* small cell lung cancer, *RCC* renal cell carcinoma

MEM were observed as follows: 15 at the trachea, 14 at the left main bronchus, 12 at the right main bronchus, 12 at the right upper lobe bronchus, five at the right intermediate bronchus, three at the right lower lobe bronchus, three at the left upper lobe bronchus, two at the left lower lobe bronchus, and one at the right middle lobe bronchus (Table 2).

**Table 2:** Location of endobronchial mass (*n* = 67)

Trachea	15
LM	14
RM	12
RUL	12
RIB	5
RLL	3
LUL	3
LLL	2
RML	1

*Abbreviations*: *LM* left main bronchus, *RM* right main bronchus, *RUL* right upper lobe bronchus, *RIB* right intermediate bronchus, *RLL* right lower lobes bronchus, *LUL* left upper lobe bronchus, *LLL* left lower lobe bronchus, *RML* right middle lobe bronchus

Post-cryotherapy complications included minor bleeding (*n* = 14), need for multiple procedures (*n* = 12), and pneumothorax (*n* = 0). There was no major bleeding. Outcomes were symptom relief (*n* = 56), improved performance status (*n* = 49), and ability to receive further chemotherapy (*n* = 43) (Table 3).

**Table 3:** Complications and outcomes after cryotherapy (*n* = 67)

Complications	
Minor bleeding	14
Major bleeding^a^	0
Multiple procedures necessity	12
Pneumothorax^a^	0
Outcomes	
Symptoms relief	56
performance status improvement	49
Received further chemotherapy	43

^a^Those listed include complications reported from literature even without occurrence in this study

Variables, including minor bleeding (odds ratio [OR] 1.2; 95% CI 0.27–5.27; *p* = 0.87), need for multiple procedures (OR 0.37; 95% CI 0.1–1.92; *p* = 0.21), symptom relief (OR 1.3; 95% CI 0.2–11.3; *p* = 0.89), performance status improved after cryotherapy (OR 3.7; 95% CI 1.2–10.7; *p* = 0.03), and ability to receive further chemotherapy after cryotherapy with improvement of performance status (OR 4.3; 95% CI 1.4–13.7; *p* = 0.02) after cryotherapy, were analyzed to confirm their respective net effects on overall survival using multiple logistic regression analysis (Table 4). After controlling for other variables, only performance status improved after cryotherapy and ability to receive further chemotherapy after cryotherapy with improvement of performance status remained the significant factors with impacting survival.

**Table 4:** Factors for survival, by multivariate analysis (*n* = 67)

	OR	95% CI	*p*
Minor bleeding	1.2	0.27–5.27	0.87
Multiple procedures necessity	0.37	0.1–1.92	0.21
Symptoms relief	1.3	0.2–11.3	0.89
Performance status improved after cryotherapy*	3.7	1.2–10.7	0.03
Ability to receive further chemotherapy after cryotherapy with improvement of performance status *	4.3	1.4–13.7	0.02

*Abbreviations*: *OR* odds ratio, *CI* confidential interval

*p < 0.05

**Fig. 1:**
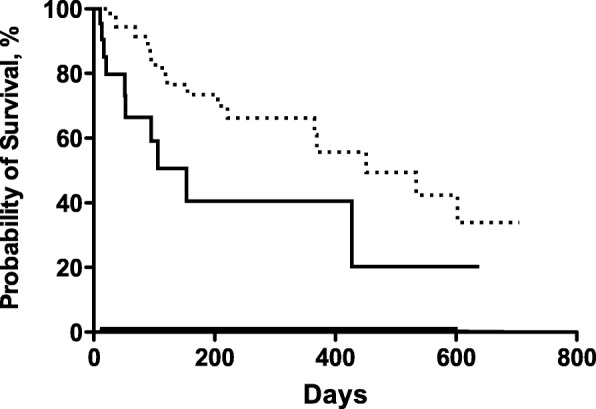
Proportion of patients having received chemotherapy and cryotherapy (………) and patients having received cryotherapy only () traced using the Kaplan-Meier method. Median, 472 vs. 169 days; log-rank test, *p*= 0.02; hazard ratio, 0.39; 95% confidence interval, 0.16-0.89

There were significant differences between patients treated with and without chemotherapy after cryotherapy. By the Kaplan-Meier method, patients having received chemotherapy and cryotherapy had better survival than patients having received cryotherapy only. (median, 472 vs. 169 days; log-rank test, *p* = 0.02; HR 0.39; 95% CI 0.16–0.89) (Fig. 1).

## Discussion

Malignant endobronchial mass could be removed by cryotherapy and chemotherapy after cryotherapy is possible. The survival improves of these patients after chemotherapy in current cohort study. However, the timing of cryotherapy for patients with central airway obstruction by tumor remains a challenging issue. All patients in this study presented with central airway obstruction (CAO) by tumor. CAO by tumor results in the poor performance due to dyspnea on exertion and even at rest. In a previous study, we addressed the diagnosis and management of CAO [16]. In this article, we explained that “There are three types of central airway tumor involvement: endoluminal (tumor within airway), extraluminal (airway narrowing from external compression), and mixed.” Cryotherapy is strongly recommended for patients with mixed or endoluminal tumors (tumor within airway). Therefore, we usually apply cryotherapy for these two types of patients. However, the degree of central airway obstruction, which causes symptoms or worsens performance status, has not been well addressed. A few reports [1, 17] have mentioned that endoluminal diameters less than 50% of normal could be suggestive of “significant airway obstruction”. However, the literature lacks clinical or psychological evidences to support that symptoms presenting during exertion may be associated with CAO unless the diameter of the central airway lumen less is than 8 mm. The symptoms at rest do not present until the diameter of central airway lumen is less than 5 mm.

The mechanism of cryotherapy improves survival of patients with MEM could be the improvement of performance status in current study. Additionally, further chemotherapy could be another factor to improve survival. However, abscopal effect of cryotherapy could be the possible mechanism. By immune modulatory effect after cryotherapy, the host immune could help the patients with better performance status and make further cancer treatment and management possible. Therefore, survival could improve by the abscopal effect. Similarly, Joe Abdo et al. [18] also reported the effect of cryotherapy in patients with cancer.

Of particular concern of the proposed treatment is bleeding and hemostasis during cryotherapy. Life-threatening bleeding is rare. During this study, no life-threatening bleeding was observed. We typically addressed any bleedingusinga bosmin flush or a scope wedge compression for minor bleeding. In the past, we also performed interventional bronchoscopy for patients with malignant diseases of the lung or airway. Electrocautery and argon plasma coagulation may be applied for major bleeding situations if bosmin flush and scope wedge compression failed to stop bleeding. Occasionally, major bleeding has happened: airway protection with the endotracheal tube remains the first priority for emergency. Recently, we applied tamponade balloons to address major bleeding. A recent report [19] also has provided recommendations for managing bleeding during flexible bronchoscopy.

The present study has limitations. First, the study was retrospective and bias, confounders and missing data could be possible. Second, it may be too simple to analyze factors to impact survival in this study. Third, in present manuscript, we enrolled patients from our register systems of patients received cryotherapy. Therefore, some of the study patients were also analyzed in previous manuscript for the diagnostic role of cryobiopsy, but the study populations of both manuscripts are not identical. The aim of previous study was to determine the diagnostic role of cryobiopsy in patients with central airway malignancy. Meanwhile, by a cohort database, the clinical outcomes of these patients remained unknown, because many of the patients were still receiving subsequent therapies against cancer. After adequate follow-up periods in these years, we could analyze the clinical outcomes of these patients. Therefore, we reported these novel results in the present manuscript.

## Conclusion

The cryotherapy could be useful management of MEM by fexlible bronchoscopy. The performance status after cryotherapy improved and caused further chemotherapy possible for the study patients and thereby, improved survival. However, the mechanism in detail of cryotherapy improve survival should be explored in the future.

## Abbreviations

CI: Confidence interval
CO_2_: Carbon dioxide
ECG: Electrocardiography
HR: Hazard ratio
IQR: Inter-quartile range
IRB: Institutional review board
LLL: Left lower lobe bronchus
LM: Left main bronchus
LUL: Left upper lobe bronchus
MEM: Malignant endobronchial mass
NSCLC: Non-small cell lung cancer
OR: Odds ratio
RCC: Renal cell carcinoma
RIB: Right intermediate bronchus
RLL: Right lower lobes bronchus
RM: Right main bronchus
RML: Right middle lobe bronchus
RUL: Right upper lobe bronchus
SCLC: Small cell lung cancer
SqCC: Squamous cell carcinoma
TB: Tuberculosis
